# Dual-Band Band-Pass Filter with Fixed Low Band and Fluidically-Tunable High Band

**DOI:** 10.3390/s17081884

**Published:** 2017-08-16

**Authors:** Eiyong Park, Daecheon Lim, Sungjoon Lim

**Affiliations:** School of Electrical and Electronics Engineering, College of Engineering, Chung-Ang University, 84 Heukseok-ro, Dongjak-gu, Seoul 06974, Korea; rntqkdl9@naver.com (E.P.); ldc2845@naver.com (D.L.)

**Keywords:** band-pass filter, dual-band, frequency tunable, microfluidic channel, liquid metal

## Abstract

In this work, we present a dual-band band-pass filter with fixed low-band resonant frequency and tunable high-band resonant frequency. The proposed filter consists of two split-ring resonators (SRRs) with a stub and microfluidic channels. The lower resonant frequency is determined by the length of the SRR alone, whereas the higher resonant frequency is determined by the lengths of the SRR and the stub. Using this characteristic, we fix the lower resonant frequency by fixing the SRR length and tune the higher resonant frequency by controlling the stub length by injecting liquid metal in the microfluidic channel. We fabricated the filter on a Duroid substrate. The microfluidic channel was made from polydimethylsiloxane (PDMS), and eutectic gallium–indium (EGaIn) was used as the liquid metal. This filter operates in two states—with, and without, the liquid metal. In the state without the liquid metal, the filter has resonant frequencies at 1.85 GHz and 3.06 GHz, with fractional bandwidths of 4.34% and 2.94%, respectively; and in the state with the liquid metal, it has resonant frequencies at 1.86 GHz and 2.98 GHz, with fractional bandwidths of 4.3% and 2.95%, respectively.

## 1. Introduction

The increasing number of multi-standard and multi-application telecommunication systems including cognitive radios [1], modern transceivers [2], anti-jamming communication systems [3], and radar systems [4], has led to the development of new tunable filter topologies [5,6]. Such system require microwave circuits and components that can control variety different frequency bands and bandwidths. To meet the requirements in a variety of areas, such as the WLAN system, the design of multi-bandpass filters with tunable frequencies is essential due to their potential to reduce system size and complexity [7,8,9].

Tunable filters can be developed electronically, using solid-state varactors or switches, microelectromechanical system (MEMS) [10,11,12,13] switches or capacitors, or variable dielectric capacitors; magnetically, using yttrium–iron–garnet (YIG) [14,15]; or by using RF switches exploiting phase-changing materials [16]. Tunable filters that use new materials such as liquid crystals (LC) or liquid metals have recently been reported. The LC tunable filter is based on the property wherein the arrangement of the LC changes when a voltage is applied [17]. In the case of filters that use liquid metals, the filters utilize the properties of the liquid metal, which possesses properties of both liquids and solids and moved by pressure alone [18]. Using these characteristics, the performance of the device has been improved in various fields, such as antennas [19], sensors [20], amplifiers [21], baluns [22], and resonators [23]. Especially, single-band tunable filters using liquid metal as a tunable device have been developed in low-pass filters and band-pass filters [24,25]. In this work, we propose a dual band tunable bandpass filter using liquid metal. In addition, the proposed filter can change only the high-band resonant frequency while fixing the low-band resonant frequency. The fixed frequency is 1.8 GHz band which can be used for wireless communications [26]. On the other hands, the tunable band can be used for sensors to detect liquid materials.

In this work, the fundamental structure of the filter employed is a dual-band band-pass filter (BPF) structure using stub-loaded resonators [27,28]. We realized the fixed-low-band and reconfigurable-high-band filter by changing the stub length, using liquid metal and microfluidic channels. The liquid metal used was eutectic gallium–indium (EGaIn), consisting of 24.5% indium and 75.5% gallium [29]. EGaIn provides advantages over other liquid metals. It has a low level of toxicity and has a thin, solid-like oxide skin on its surface to improve mechanical stability [30]. The microfluidic channels used as the paths for the liquid metal were made of polydimethylsiloxane (PDMS) elastomer and 3D-printed frames [31]. The performance of the proposed BPF is validated from both simulation and measurements.

## 2. Frequency-Tunable Band-Pass Filter Design

**Figure 1:**
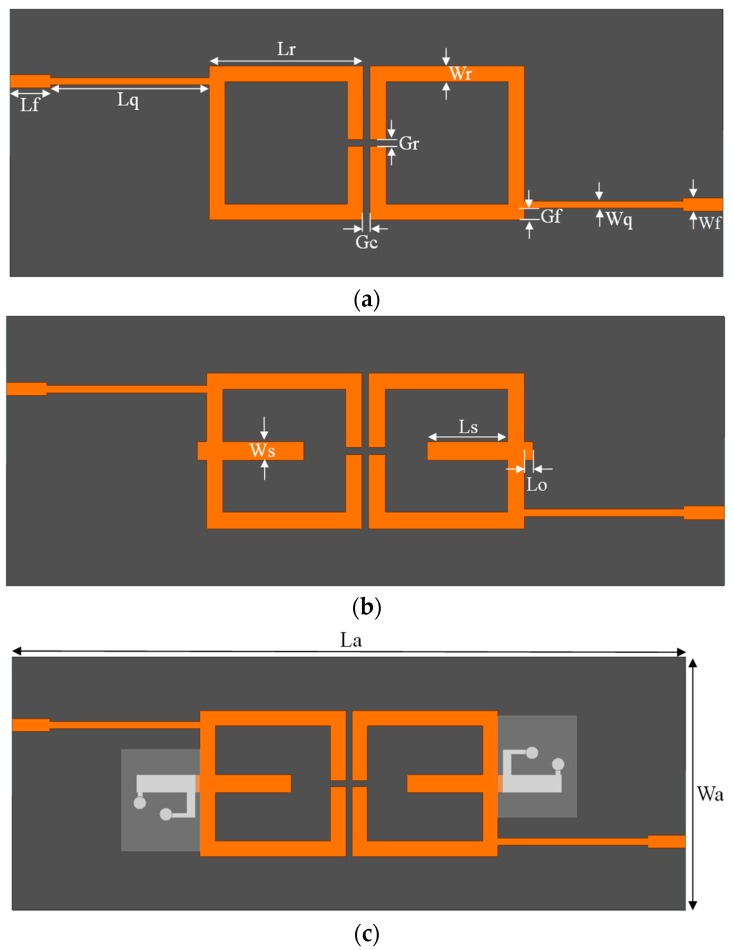

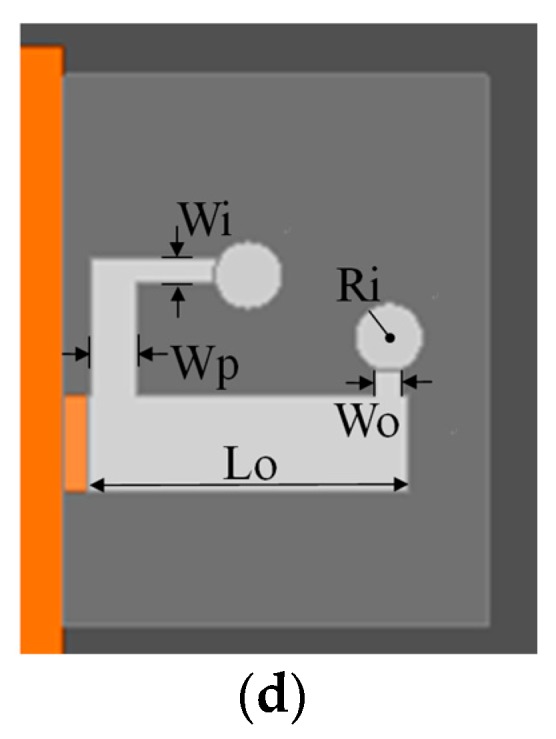
Top view of the proposed filter: (**a**) using two split-ring resonators (SRR); (**b**) with stub; (**c**) with stub and microfluidic channel; and (**d**) an enlarged top view of the microfluidic channel.

In this paper, we present a dual-band BPF with a fixed low-band resonant frequency and a fluidically-tunable high-band resonant frequency. The design of the proposed BPF is based on a coupled resonator filter that uses two split-ring resonators, as shown in Figure 1a. To realize the dual-band BPF, we add a stub to the coupled resonator filter, as shown in in Figure 1b. Finally, we add a microfluidic channel to the coupled-resonator filter with stub to create the frequency-controllable filter shown in Figure 1c. The detailed design of the microfluidic channel for frequency switching, which is created using polydimethylsiloxane (PDMS), is shown in Figure 1d. The values of the parameters in Figure 1 are *L_a_* = 80, *L_r_* = 17.3, *L_f_* = 4.492, *L_q_* = 17.823, *L_s_* = 9, *L_o_* = 1, *L_c_* = 7.065, *W_a_* = 30, *W_r_* = 1.8, *W_q_* = 0.83, *W_f_* = 1.5, *W_s_* = 2.1, *W_p_* = 1, *W_i_* = 0.55, *W_o_* = 0.6, *G_r_* = 0.77, *G_c_* = 0.77, and *R_(SA (θ))_* = 0.75 [mm].

**Figure 2:**
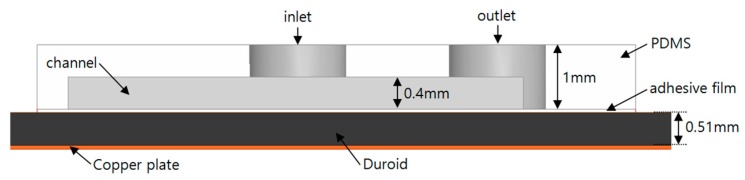
Side view of the proposed filter.

Figure 2 shows the side view of the proposed filter. The proposed filter consists of a copper plate, Duroid substrate, adhesive film, and PDMS. The bottom layer is the copper plate, which acts as the ground. The substrate for the proposed filter is a 0.51 mm thick Duroid 5880 substrate (Rogers, Killingly, CT, USA), with a permittivity of 2.2. The adhesive film bonds the Duroid substrate and the PDMS layer. We used 0.05 mm thick ARcare^®^ 92561 (Adhesives Research, Glen Rock, PA, USA) for the adhesive film which can be simply attached on the PCB substrate without any post processing. PDMS, with a permittivity of 3.2, is used to create the microfluidic channel. The resonant frequency of the proposed filter is divided into two types of modes. The lower one is the odd mode and the other is the even mode. The odd-mode frequency (*f_odd_*) is related to the length of the split-ring resonator, which is *L_r_* in Figure 1a. The relationship between the odd-mode frequency (*f_odd_*) and *L_r_* is given by:

(1) $$f_{odd}=\frac{(2n-1)c}{8L_{r}\sqrt{\varepsilon_{eff}}}$$

where *c* is the velocity of light and *n* is a positive integer and *ε_eff_* is the effective permittivity of the substrate.

The even-mode frequency is related to the length of the split-ring resonator and the length of the stub; that is, the sum of *L_s_* and *L_o_* in Figure 1b. The relationship between the even-mode frequency (*f_even_*) and *L_r_*, *L_s_*, and *L_o_* is given by:

(2) $$f_{even}=\frac{nc}{(4L_{r}+2L_{s}+2L_{o})\sqrt{\varepsilon_{eff}}}$$

**Figure 3:**
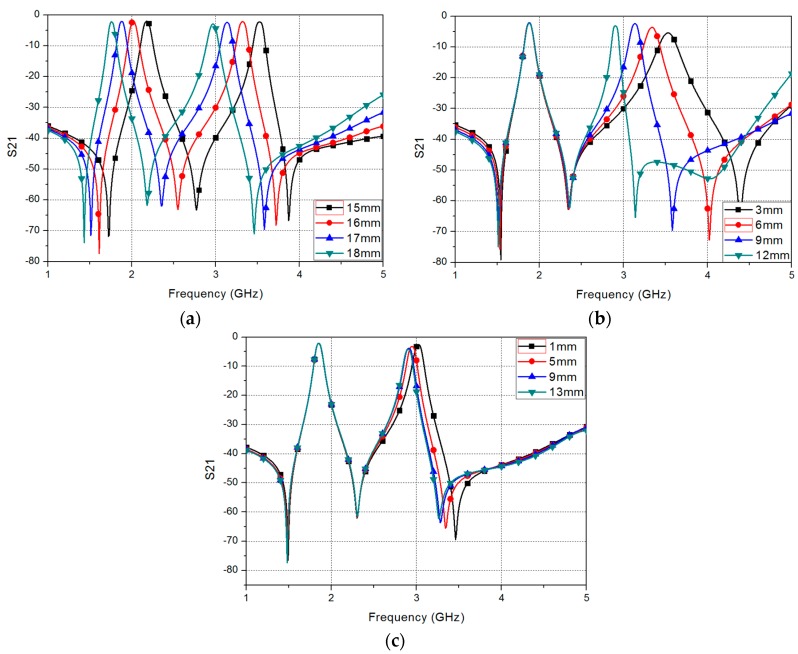
Simulated S_21_ of the proposed filter for: (**a**) different *L_r_*; (**b**) different *L_s_*; and (**c**) different *L_c_*.

Figure 3 shows the simulation results of the proposed filter for different parameters. Figure 3a shows the insertion loss (S_21_) of the proposed filter, for different *L_r_*. When *L_r_* is increased, the odd frequency and even frequency are decreased according to Equations (1) and (2), respectively. Figure 3b shows the S_21_ of the proposed filter, for different *L_s_*. When *L_s_* is increased, the even-mode frequency is decreased, but the odd-mode frequency is not changed, according to Equations (1) and (2). Using this characteristic, the proposed filter can fix the odd frequency and change the even frequency by controlling the length of the stub. To control the length of the stub, we add a microfluidic channel to the stub of the filter. Due to the limited space inside the SRR, we loaded the microfluidic channel to change *L_o_* instead of *L_s_*. Therefore, we can minimize electromagnetic coupling. When liquid metal is injected into the microfluidic channel, the length of the stub is increased depending on the length of the microfluidic channel, which is given by *L_c_* in Figure 1d. Figure 3c shows the S_21_ of the proposed filter, for different *L_c_*, when liquid metal is injected into the microfluidic channel. When *L_c_* is increased, the odd frequency shows no change, but the even frequency is decreased.

**Figure 4:**
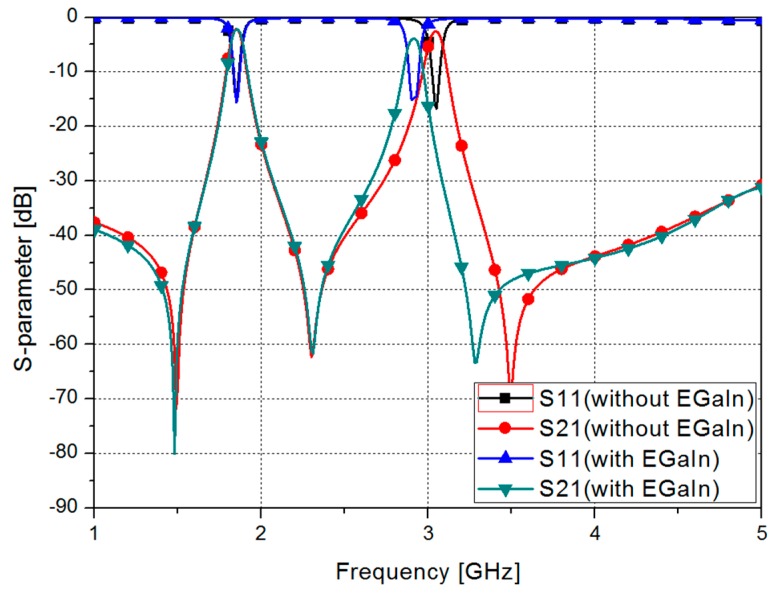
Simulated S-parameters of the proposed filter.

The S-parameters of the proposed filter are shown in Figure 4, for the different cases where liquid metal is injected and not injected into the microfluidic channel. The odd-mode frequency of the proposed filter without liquid metal is 1.85 GHz, and the fractional bandwidth is 4.32%. The even-mode frequency of the proposed filter without liquid metal is 3.05 GHz, and the fractional bandwidth is 2.96%. The odd-mode frequency of the proposed filter with liquid metal is 1.85 GHz, and the fractional bandwidth is 4.32%. The even-mode frequency of the proposed filter with liquid metal is 2.9 GHz, and the fractional bandwidth is 2.75%. From these results, it can be inferred that, when liquid metal is injected into the microfluidic channel, the odd-mode frequency is not changed, while the even-mode frequency is shifted by approximately 150 MHz.

The fractional bandwidth is defined by the following equation [32]:

(3) $$\frac{f_{fh}-f_{fl}}{f_{c}}\times 100\%$$

where *f_fh_* and *f_fl_* are higher and lower 3 dB frequency and *f_c_* is center frequency.

## 3. Fabrication and Measurement

**Figure 5:**
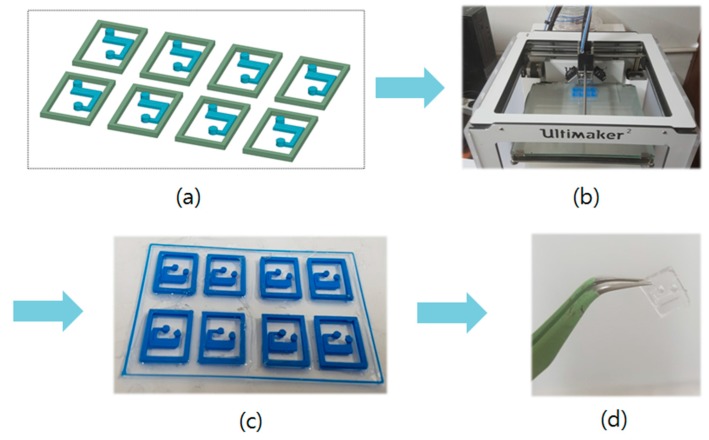
Process of fabricating the microfluidic channel: (**a**) design; (**b**) 3D printing; (**c**) PDMS solidification; and (**d**) the fabricated microfluidic channel.

Figure 5 shows the fabrication of the PDMS microfluidic channel. First, we make a mold for the PDMS microfluidic channel, before fabricating it, because we cannot fabricate the PDMS channel using a 3D printer alone. To make the mold for the channel, we design a mold using a 3D modeler program, as shown in Figure 5a. The designed mold is realized using a 3D printer (Ultimaker2, Geldermalsen, The Netherlands), as shown in in Figure 5b. Then, we fabricate the PDMS channel using the mold. Figure 5c shows the fabricated mold. We create PDMS by solidifying the liquid made by mixing Sylgard184 Base and Sylgard184 Agent in the ratio of 10:1. We then fabricate the PDMS channel by pouring the mixed liquid into the mold and solidifying by heating for 30 min. Figure 5d shows the fabricated microfluidic channel.

**Figure 6:**
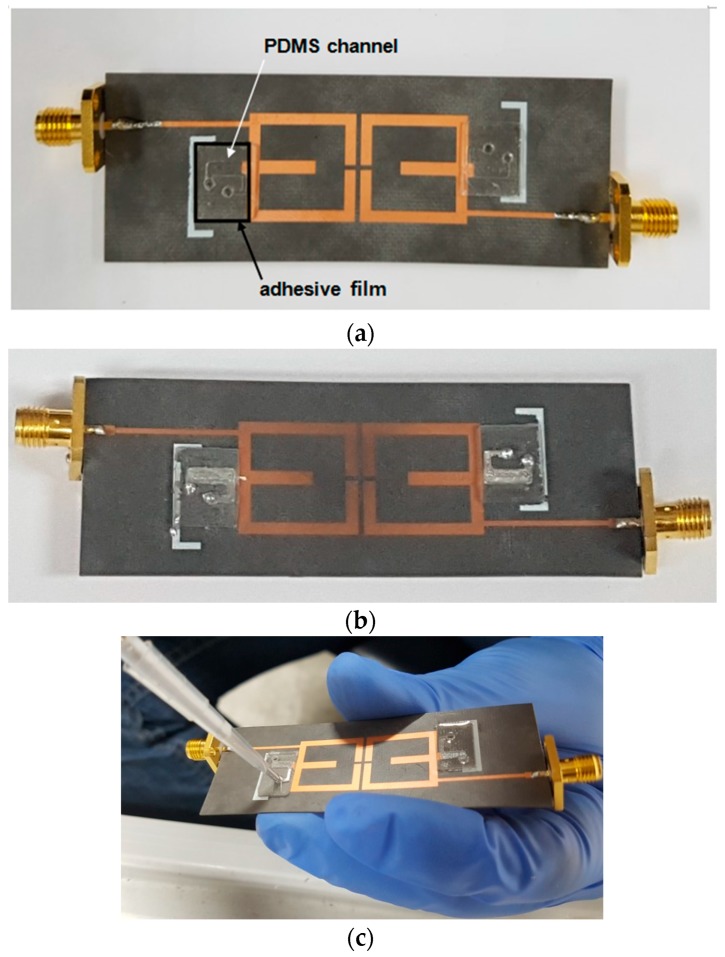
Pictures of the fabricated proposed filter: (**a**) without EGaIn; (**b**) with EGaIn; and (**c**) in the process of injecting EGaIn.

Figure 6a,b shows pictures of samples of the fabricated proposed filter. The substrate is realized using Duroid 5880 board (Rogers, Killingly, CT, USA). The patterns on the top and ground of the sample are realized using copper. Microfluidic channels made by the process in Figure 5 are attached onto the pattern and substrate. The difference between Figure 6a,b is the liquid metal is not injected in the microfluidic channel in Figure 6a and injected in the microfluidic channel in Figure 6b. We use eutectic gallium–indium (EGaIn) for the liquid metal. For injecting EGaIn into the microfluidic channel and extracting EGaIn from the microfluidic channel, we use an injector named Pipetman, shown in Figure 6c.

Table 1 shows comparisons of the measured and simulated result of the proposed filter. The S-parameters of the filter are measured using an Anritsu MS2038C. When the proposed filter without EGaIn, the odd-mode frequency of the proposed filter is 1.85 GHz, and is the same in both the results. The even-mode frequency of the measured result is 3.06 GHz, and the even-mode frequency of the simulation result is 3.05 GHz, which show a very small difference. When the proposed filter with EGaIn, the odd-mode frequency of the measured result is 1.85 GHz, and the odd-mode frequency of the simulation result is 1.86 GHz. The even-mode frequency of the measured result is 2.98 GHz, whereas the even-mode frequency of the simulation result is 2.9 GHz.

The measurement resonant frequency with EGaIn is slightly higher than the simulated resonant frequencies. As shown in Figure 3c, the insertion loss is increased with lower resonant frequency because of larger EGaIn. Therefore, the measured insertion loss with EGaIn is higher than the simulated insertion loss with EGaIn.

**Figure 7:**
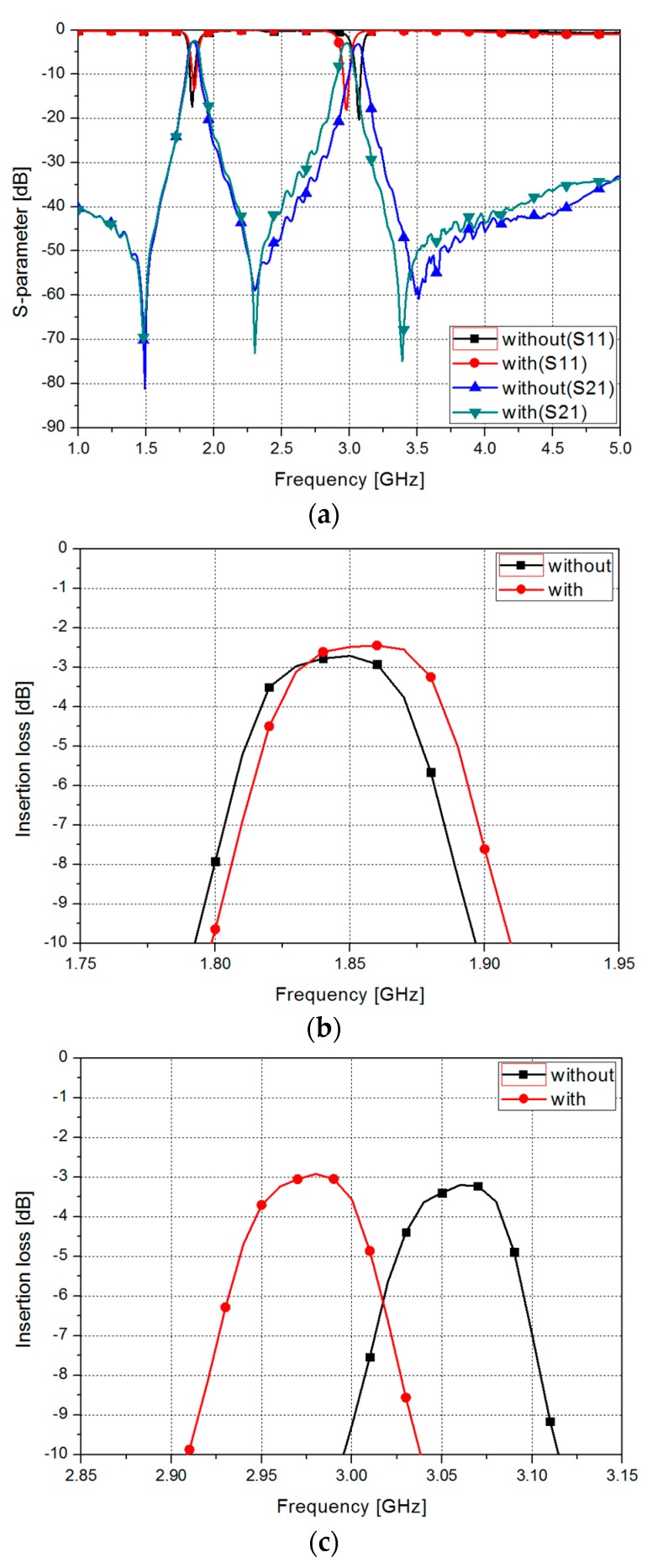
(**a**) Measured S-parameters of the proposed filter without and with EGaIn and insertion loss without and with EGaIn (**b**) in odd mode; and (**c**) in even mode.

Figure 7 shows the measured results of the S-parameters of the proposed filter without and with EGaIn. For the odd mode, the filter without EGaIn has the insertion loss of 2.72 dB at 1.85 GHz and a fractional bandwidth of 4.34%. The filter with EGaIn has the insertion loss of 2.5 dB at 1.86 GHz and a fractional bandwidth of 4.3%.

In this graph, when EGaIn is injected into the microfluidic channel, the odd-mode frequency and fractional bandwidth are not changed. For the even mode, the filter without EGaIn has the insertion loss of 3.21 dB at 3.06 GHz and a fractional bandwidth of 2.94%. The filter with EGaIn has the insertion loss of 2.5 dB at 2.98 GHz and a fractional bandwidth of 2.95%.

In this graph, when EGaIn is injected into the microfluidic channel, the even-mode frequency is shifted by 0.11 GHz and the fractional bandwidth is slightly decreased.

## 4. Conclusions

In this paper, we proposed a dual-band BPF with fixed low-band resonant frequency and fluidically-tunable high-band frequency. The filter consisted of two split-ring resonators, a stub for providing dual-band operation, and a microfluidic channel for tuning the frequency. The proposed filter was based on the characteristic that the lower resonant frequency was determined by the length of the SRR and the higher resonant frequency by the lengths of the SRR and the stub. We controlled the length of the stub using a microfluidic channel and EGaIn, to fix the lower-band frequency and tune the higher-band frequency. The filter was fabricated on a Duroid 5880 substrate and the microfluidic channel was fabricated using a 3D Printer. From the measurement results, the lower band frequency was found to be fixed at 1.85 GHz and the higher band frequency was found to be shifted from 3.06 GHz to 2.95 GHz, when EGaIn was injected into the microfluidic channel.

In Table 2, we compared the performances of the proposed filters with those of other tunable filters using liquid metal. Although insertion losses is higher than other filters, the propose filter operates at dual band. In addition, only high band resonant frequency can be controlled while fixing the low band resonant frequency. The tuning range is defined by the following equation:

(4) $$\mathrm{Tuning}\;\mathrm{Range}\;=\;\frac{f_{high}-f_{low}}{f_{high}}\times 100\;[\%]$$

where *f_low_* and *f_high_* are lowest and highest resonant frequencies, respectively. The tuning range can be increased by increasing the length of the microfluidic channel and injecting more liquid metal in the microfluidic channel.

## Figures and Tables

**Table 1:** Summary of simulated and measured results of the proposed filter.

		Without EGaIn		With EGaIn	
		Simulation	Measurement	Simulation	Measurement
Odd Mode	Resonant Freuqnecy (GHz)	1.85	1.85	1.85	1.86
	Insertion Loss (dB)	2.15	2.72	2.17	2.45
	Fractional Bandwidth (%)	4.32	4.34	4.32	4.3
Even Mode	Resonant Freuqnecy (GHz)	3.05	3.06	2.9	2.98
	Insertion Loss (dB)	2.57	3.21	3.9	2.93
	Fractional Bandwidth (%)	2.75	2.94	2.96	2.95

**Table 2:** Comparison table of the proposed filter performance with other liquid metal tunable filters.

	[18]	[24]	[25]	Proposed Work
Filter type	Lowpass	Band-pass	Band-pass	Dual Band-pass
Insertion Loss (dB)	N/A	<3	<1.5	<2.72, <3.21
Bandwidth (%)	N/A	5	9.38	4.34, 2.95
Tuning Range (%)	38	25.3	14	2.7
Number of band	Single Band	Single Band	Signle Band	Dual Band
